# Genetic Analysis of Virulence Potential of *Escherichia coli* O104 Serotypes Isolated From Cattle Feces Using Whole Genome Sequencing

**DOI:** 10.3389/fmicb.2018.00341

**Published:** 2018-03-01

**Authors:** Pragathi B. Shridhar, Isha R. Patel, Jayanthi Gangiredla, Lance W. Noll, Xiaorong Shi, Jianfa Bai, Christopher A. Elkins, Nancy A. Strockbine, T. G. Nagaraja

**Affiliations:** ^1^Department of Diagnostic Medicine and Pathobiology, Kansas State University, Manhattan, KS, United States; ^2^Division of Molecular Biology, Center for Food Safety and Applied Nutrition, United States Food and Drug Administration, Laurel, MD, United States; ^3^Veterinary Diagnostic Laboratory, Kansas State University, Manhattan, KS, United States; ^4^Division of Foodborne, Waterborne, and Environmental Diseases, National Center for Emerging and Zoonotic Infectious Diseases, Centers for Disease Control and Prevention, Atlanta, GA, United States

**Keywords:** *E. coli* O104, cattle feces, serotypes, whole genome sequencing, virulence genes

## Abstract

*Escherichia coli* O104:H4, a Shiga toxin-producing hybrid pathotype that was implicated in a major foodborne outbreak in Germany in 2011, has not been detected in cattle. However, serotypes of O104, other than O104:H4, have been isolated from cattle feces, with O104:H7 being the most predominant. In this study, we investigated, based on whole genome sequence analyses, the virulence potential of *E. coli* O104 strains isolated from cattle feces, since cattle are asymptomatic carriers of *E. coli* O104. The genomes of ten bovine *E. coli* O104 strains (six O104:H7, one O104:H8, one O104:H12, and two O104:H23) and five O104:H7 isolated from human clinical cases were sequenced. Of all the bovine O104 serotypes (H7, H8, H12, and H23) that were included in the study, only *E. coli* O104:H7 serotype possessed Shiga toxins. Four of the six bovine O104:H7 strains and one of the five human strains carried *stx*1c. Three human O104 strains carried *stx*2, two were of subtype 2a, and one was 2d. Genomes of *stx* carrying bovine O104:H7 strains were larger than the *stx*-negative strains of O104:H7 or other serotypes. The genome sizes were proportional to the number of genes carried on the mobile genetic elements (phages, prophages, transposable elements and plasmids). Both bovine and human strains were negative for intimin and other genes associated with the type III secretory system and non-LEE encoded effectors. Plasmid-encoded virulence genes (*ehxA, epeA, espP, katP*) were also present in bovine and human strains. All O104 strains were negative for antimicrobial resistance genes, except one human strain. Phylogenetic analysis indicated that bovine *E. coli* O104 strains carrying the same flagellar antigen clustered together and STEC strains clustered separately from non-STEC strains. One of the human O104:H7 strains was phylogenetically closely related to and belonged to the same sequence type (ST-1817) as the bovine O104:H7 STEC strains. This suggests that the bovine feces could be a source of human illness caused by *E. coli* O104:H7 serotype. Because bovine O104:H7 strains carried virulence genes similar to human clinical strains and one of the human clinical strains was phylogenetically related to bovine strains, the serotype has the potential to be a diarrheagenic pathogen in humans.

## Introduction

Shiga toxin-producing *Escherichia coli* (STEC) O157 and six serogroups of non-O157 STEC, O26, O45, O103, O111, O121, and O145, are responsible for foodborne illnesses that range from mild to bloody diarrhea to serious complications of hemolytic uremic syndrome (HUS) in humans (Gould et al., 2013). Cattle are a major reservoir of O157 and non-O157 STEC serogroups, which harbor the organisms in their hindgut and shed them in the feces. The “top 7” STEC have been declared as adulterants in ground beef and non-intact raw beef products by the U.S. Department of Agriculture, Food Safety and Inspection Service (USDA-FSIS, 2011). In 2011, a novel serotype, *E. coli* O104:H4, was responsible for a large outbreak of hemorrhagic colitis and HUS in humans in Germany. The serotype was a hybrid pathotype carrying genes characteristic of enteroaggregative *E. coli* (EAEC) and STEC (Bielaszewska et al., 2011). Another serotype, *E. coli* O104:H21, was responsible for a small outbreak of hemorrhagic colitis in Helena, Montana in 1994 linked to consumption of contaminated milk (CDC, 1995). In addition, there are reports of sporadic cases of diarrhea associated with *E. coli* O104:H2, O104:H7, and O104:H12 (Delannoy et al., 2012a; Miko et al., 2013).

The German outbreak strain of *E. coli* O104:H4 has not been detected in cattle feces (Wieler et al., 2011; Auvray et al., 2012; Paddock et al., 2013; Shridhar et al., 2016). However, cattle do harbor and shed other serotypes of O104; including O104:H2, O104:H7, O104:H11, and O104:H21 (Paddock et al., 2013; Shridhar et al., 2016). Of the serotypes, O104:H7 appeared to be the dominant serotype and the only serotype that carried Shiga toxin gene (*stx*1) (Shridhar et al., 2016).

Shiga toxins (Stx) are major virulence factors of STEC and are responsible for life-threatening complications, such as HUS, associated with STEC infections (Karmali et al., 1985). Intimin, encoded by *eae*, a key adhesin expressed by enterohemorrhagic (EHEC), a subset of STEC, and enteropathogenic *E. coli* (EPEC), is responsible for the intimate attachment of bacteria to the host epithelial cells leading to attachment and effacement lesion (DeVinney et al., 1999). In addition, a number of other virulence factors, encoded by genes located on the chromosome or mobile genetic elements, are also involved in the pathogenesis of *E. coli* infections (Kaper et al., 1999). Therefore, determining the virulence gene profiles of *E. coli* O104 serotypes isolated from cattle is required to assess their potential to cause human illness. Whole genome sequence analysis provides a greater insight into the virulence potential and genetic diversity of strains within a serotype. The objectives of this study were to determine the virulence genes repertoire of *E. coli* O104 strains isolated from cattle by whole genome sequence analysis and to determine their phylogenetic relationship. Human clinical strains of *E. coli* O104:H7 were included for comparison.

## Materials and methods

### *Escherichia coli* O104 strains

Ten *E. coli* O104 strains isolated from rectal contents of feedlot cattle collected at a slaughter plant (Shridhar et al., 2016) and five *E. coli* O104 strains isolated from sporadic cases of diarrhea from human patients (Centers for Disease Control and Prevention, Atlanta, GA) were included in the study. Details on the study population, sampling and isolation procedures of cattle *E. coli* O104 strains have been described (Shridhar et al., 2016). Of the 10 cattle strains, six were identified to contain H7 flagellar antigen (Shridhar et al., 2016) and the other four were of unknown serotypes. All five human strains carried H7 flagellar type. The strains were cultured onto Tryptone soy agar (TSA; BD Difco, Sparks, MD) slants and shipped overnight to the Center for Food Safety and Applied Nutrition, Food and Drug Administration, Laurel, MD, for whole genome sequencing.

### DNA extraction and whole genome sequencing

*Escherichia coli* O104 strains were streaked onto blood agar (Remel, Lenexa, KS). A single colony of each strain was inoculated into Luria Bertani (LB) broth and incubated on a shaker at 37°C. Genomic DNA was isolated from overnight culture using the Qiagen DNeasy blood and tissue kit (Qiagen, Inc., Valencia, CA). The purity of the DNA was assessed spectrophotometrically using the Nanodrop (Thermo Scientific, Waltham, MA). Genomic libraries of the strains were constructed using Nextera XT DNA Library Preparation kit and whole genome sequencing was performed on an Illumina MiSeq benchtop sequencer (Illumina, Inc., San Diego, CA) using the MiSeq version 2 reagent kit with 2 × 250 cycles. *De novo* assembly of quality-controlled trimmed sequenced reads was performed using the SPAdes genome assembler version 3.8.2 (http://cab.spbu.ru/software/spades/) (Bankevich et al., 2012).

### Sequence analysis

Draft genomes of *E. coli* O104 strains were initially annotated using RAST (Rapid Annotation using Subsystem Technology) server (Aziz et al., 2008). The RAST server also provides data on the distribution of genes in various categories. Serotypes, virulence genes and antimicrobial resistance genes of the strains were determined using SerotypeFinder 1.1 (Joensen et al., 2015), VirulenceFinder 1.5 (Joensen et al., 2014), and ResFinder 2.1 (Zankari et al., 2012), respectively, which are web-based tools developed by the Center for Genomic Epidemiology (CGE) at the Danish Technical University (DTU), Lyngby, Denmark (http://www.genomicepidemiology.org/). Different types of plasmid sequences were identified by PlasmidFinder 1.3 (https://cge.cbs.dtu.dk/services/PlasmidFinder/) tool (Carattoli, 2009). The total number of prophage sequences were determined using Phage Search Tool Enhanced Release (PHASTER; http://phaster.ca/). The tool identifies intact, questionable, and incomplete prophage sequences by scores of >90, 70–90, < 70, respectively (Zhou et al., 2011; Arndt et al., 2016). Clusters of regularly interspaced short palindromic repeats (CRISPR)-Cas system of bovine and human *E. coli* O104 strains were characterized based on annotation by CRISPRone, a web-based tool (http://omics.informatics.indiana.edu/CRISPRone). The tool provides class, type, and subtype of CRISPR-Cas system and number, length and nucleotide sequences of repeats and spacers (Zhang and Ye, 2017). The sequence types of the strains were determined *in silico* using MLST 1.8 (https://cge.cbs.dtu.dk/services/MLST/) (Wirth et al., 2006; Jaureguy et al., 2008). The Harvest Suite, a software package which includes tools such as Parsnp and Gingr, was used to determine the phylogenetic relationship among the *E. coli* O104 strains (Treangen et al., 2014). Parsnp v1.2 (http://harvest.readthedocs.io/en/latest/content/parsnp.html) was used to align the core genomes of human and bovine *E. coli* O104 strains, followed by the construction of maximum likelihood tree. *Escherichia coli* O104:H21, a human outbreak strain (Montana; GenBank accession no. CP009106.2) was also included in the phylogenetic analysis to determine the genetic relatedness to the bovine O104 strains. The phylogenetic tree was subsequently imported to FigTree 1.4.3 software (http://tree.bio.ed.ac.uk/software/figtree/) (Rambaut, 2016) for better visualization, and bootstrap values are reported for each branch.

### Statistical analysis

A single factor analysis of variance (ANOVA) test was performed to determine whether genome size and number of genes associated with different functional categories were significantly different between bovine STEC, non-STEC and human strains. If the means were significantly different (*P* < 0.01), then Tukey adjustment for multiple comparisons was performed using PROC GLIMMIX procedure of SAS version 9.4 (SAS Institute Inc., Cary, NC), to test each pairwise comparison.

## Results

Serotypes of bovine (*n* = 6) and human O104:H7 (*n* = 5) strains were confirmed by SerotypeFinder 1.1 and the other four unknown serotypes from cattle were identified as O104:H8, O104:H12, and 104:H23 (*n* = 2).

### Rapid annotation using subsystem technology

The genome size ranged from 5.2 to 5.3 Mb, and 4.7 to 5.0 Mb for STEC and non-STEC bovine strains, respectively. The genome size of human *E. coli* O104 strains ranged from 4.9 to 5.4 Mb. The average genome size of bovine STEC strains (5.3 Mb) were significantly (*P* < 0.01) larger than bovine non-STEC strains (4.9 Mb). The functional categorization of genes revealed that the number of genes associated with virulence, disease, and defense ranged from 109 to 116 in bovine strains and 110 to122 in human O104 strains. The number of genes carried on mobile elements such as phages, prophages, transposable elements and plasmids ranged from 60 to 201 in bovine O104 strains, and 80 to 201 in human O104 strains. The average number of genes associated with mobile genetic elements was significantly (*P* < 0.01) higher in bovine STEC strains (190) compared to non-STEC strains (71). The human strains had lower (*P* < 0.01) number of genes associated with mobile genetic elements than bovine O104:H7 STEC strains. There was no significant difference in the average number of genes associated with other subsystem categories (membrane transport, iron acquisition and metabolism, and stress response) between bovine and human strains (Table 1).

**Table 1:** Genome size and total number of major categories of genes in *E. coli* O104 strains isolated from cattle feces and human clinical cases^#^.

**Strains**	**Serotype**	***stx***	**Genome size (Mb)**	**Virulence, disease, and defense**	**Phages, prophages, transposable elements and plasmids**	**Membrane transport**	**Iron acquisition and metabolism**	**Stress response**
**CATTLE**								
2013-6-685A	O104:H7	+	5.2	109	188	221	23	187
2013-6-48C	O104:H7	+	5.3	109	201	223	24	184
2013-6-122E	O104:H7	+	5.3	109	184	223	24	186
2013-6-148B	O104:H7	+	5.3	109	186	222	23	186
2013-6-193B	O104:H7	−	4.7	109	70	195	22	183
2013-6-289D	O104:H7	−	5.0	109	65	294	22	187
2013-6-380B	O104:H8	−	5.0	116	64	216	24	186
2013-6-210A	O104:H12	−	4.8	113	60	204	26	193
2013-6-140D	O104:H23	−	5.0	111	82	237	22	184
2013-6-173D	O104:H23	−	5.0	111	85	238	22	184
**HUMAN**								
06-3637	O104:H7	+	5.0	112	95	328	22	185
08-4061	O104:H7	+	5.0	110	108	220	22	184
2011C-3665	O104:H7	+	5.4	122	201	316	22	187
2012C-3400	O104:H7	+	5.0	110	120	222	22	185
07-3598	O104:H7	−	4.9	110	80	254	22	184

# *Based on annotation by Rapid annotation using subsystem Technology (RAST) (Aziz et al., 2008)*.

### Virulence genes

Four of the six bovine and one of the five human O104:H7 strains carried *stx*1 gene of subtype c. Three human STEC O104:H7 strains carried *stx*2, two were of subtype 2a and one was 2d (Table 2). The *ast*A gene that codes for *E. coli* heat stable enterotoxin 1 was present in one bovine strain (2013-6-173D that belonged to the O104:H23 serotype). The gene *sub*A (subtilase toxin subunit) was present in 4 of 5 human strains of O104:H7, but absent in all bovine strains. Both bovine and human O104 strains were negative for intimin (*eae*) and other genes associated with LEE Pathogenicity Island, and non-LEE encoded effectors. However, both bovine and human *E. coli* O104 strains carried other adhesins, such as *irg*A homolog adhesin (*iha*) and long polar fimbriae (*lpfA*).

**Table 2:** Virulence genes and antimicrobial resistance genes present in *E. coli* O104 strains isolated from cattle feces (*n* = 10) and human clinical cases (*n* = 5)^#^.

**Genes**	**Product**	**Bovine**						**Human**					**Bovine**			
		**O104:H7 (2013-6-685A)**	**O104:H7 (2013-6-48C)**	**O104:H7 (2013-6-122E)**	**O104:H7 (2013-6-148B)**	**O104:H7 (2013-6-193B)**	**O104:H7 (2013-6-289D)**	**O104:H7 (06-3637)**	**O104:H7 (08-4061)**	**O104:H7 (2011C-3665)**	**O104:H7 (2012C-3400)**	**O104:H7 (07-3598)**	**O104:H8 (2013-6-380B)**	**O104:H12 (2013-6-210A)**	**O104:H23 (2013-6-140D)**	**O104:H23 (2013-6-173D)**
**TOXINS**																
*stx*1c	Shiga toxin 1 subtype c	+	+	+	+					+						
*stx*2a	Shiga toxin 2 subtype a								+		+					
*stx*2d	Shiga toxin 2 subtype d							+								
*subA*	Subtilase toxin subunit							+	+		+	+				
*ehxA*	Enterohemolysin	+	+	+	+			+	+			+				
*astA*	EAEC heat-stable enterotoxin 1															+
**ADHESINS**																
*iha*	*Irg*A homologue adhesin	+	+	+	+			+	+		+	+				
*lpfA*	Long polar fimbriae	+	+	+	+	+	+	+	+	+	+	+	+		+	+
**OTHERS**																
*aaiC*	Type VI secretion protein	+	+	+	+					+						
*gad*	Glutamate decarboxylase	+	+	+	+	+	+	+	+	+	+	+	+	+	+	+
*iss*	Increased serum survival	+						+	+		+	+		+		
*epeA*	Enterohemorrhagic *E. coli* plasmid-encoded autotransporter	+								+						
*cba*	Colicin B				+				+	+						
*celb*	Endonuclease colicin E2							+		+		+				
*espP*	Extracellular serine protease plasmid-encoded							+	+		+	+				
*cma*	Colicin M									+						
*katP*	Plasmid-encoded catalase peroxidase									+						
*pic*	Serine protease autotransporters of *Enterobacteriaceae* (SPATE)					+										
**ANTIMICROBIAL RESISTANCE GENES**																
*tet*	Tetracycline resistance									+						
*aadA1*	Aminoglycoside resistance									+						
*sul1*	Sulfonamide resistance									+						

# *Virulence gene profile was determined using VirulenceFinder 1.4 (Joensen et al., 2014)*.

Plasmid-encoded virulence genes (*ehx*A, *epe*A, *esp*P, *kat*P) were present in bovine and human strains. Four of the bovine strains, and three of the human strains carried *ehx*A (enterohemolysin). Enterohemorrhagic *E. coli* plasmid-encoded autotransporter (*epe*A) was present in one of the bovine strains (O104:H7; 2013-6-685A), and one human strain (O104:H7; 2011C-3665). Extracellular serine protease (*esp*P; 4/5) and catalase peroxidase (*kat*P; 1/5) were present only in human strains.

All bovine and human strains carried *gad* (Glutamate decarboxylase). Four bovine O104:H7 strains (2013-6-48C, 2013-6-122E, 2013-6-148B, and 2013-6-685A) and one human O104:H7 strain (2011C-3665) was positive for *aai*C (Type VI secretory system protein). The gene encoding increased serum survival (*iss*) was present in two bovine (2013-6-685A and 2013-6-210A) and four human O104:H7 strains (06-3637, 07-3598, 08-3046, and 2012C-3400). One of the bovine strains (O104:H7; 2013-6-193B) carried *pic* (protein involved in intestinal colonization). Different types of genes that code for colicin, a bacteriocin, were also present in bovine and human *E. coli* O104 strains. The colicin B gene (*cba*) was present in one bovine strain (2013-6-148B) and two of the human strains (08-4061 and 2011C-3665), *celb* (endonuclease colicin E2) was present in three human strains (06-3637, 07-3598, and 2011C-3665), *cma* (Colicin M) was present only in one human strain (2011C-3665). Virulence gene profiles of bovine and human strains are provided in Table 2.

### Antimicrobial resistance genes

Antimicrobial resistance genes, *tet* (tetracycline resistance), *aad*A1 (aminoglycoside resistance), and *sul*1 (sulfonamide resistance), were present only in one of the human strains (2011C-3665). All the bovine and other human strains were negative for antimicrobial resistance genes (Table 2).

### Plasmid and prophage sequences

All the bovine STEC and one (2013-6-289D) of the non-STEC O104:H7 strains carried IncFIB and IncFII plasmid sequences (Table 3). IncH12 and IncH12A plasmid sequences were carried by all except one bovine non-STEC strain (O104:H7; 2013-6-193B). IncY was present in one (O104:H8; 2013-6-380B) of the bovine non-STEC strains and one human STEC strain (08-4061). IncB/O/K/Z and IncFIB plasmid sequences were present in all human strains except one (O104:H7; 2011C-3665). Col156 was present in two human STEC and one non-STEC strains. A complete list of plasmid sequences found in bovine and human *E. coli* O104 strains is provided in Table 3.

**Table 3:** Plasmid types in *E. coli* O104 strains isolated from cattle feces and human clinical cases using PlasmidFinder 1.3.

**Serotypes**	**Strains**	***stx***	**Plasmid types^#^**						
			**IncFIB**	**IncFII**	**IncB/O/K/Z**	**Col156**	**IncY**	**IncH12**	**IncH12A**
**BOVINE**									
O104:H7	2013-6-685A	1c	+	+					
O104:H7	2013-6-48C	1c	+	+					
O104:H7	2013-6-122E	1c	+	+					
O104:H7	2013-6-148B	1c	+	+					
O104:H7	2013-6-193B	-							
O104:H7	2013-6-289D	-	+	+				+	+
**HUMAN**									
O104:H7	06-3637	2d	+		+	+			
O104:H7	08-4061	2a	+		+		+		
O104:H7	2011C-3665	1c		+		+			
O104:H7	2012C-3400	2a	+		+				
O104:H7	07-3598	-	+		+	+			
**BOVINE**									
O104:H8	2013-6-380B	-					+	+	+
O104:H12	2013-6-210A	-						+	+
O104:H23	2013-6-140D	-						+	+
O104:H23	2013-6-173D	-						+	+

# *Different types of plasmid sequences were identified using Plasmid Finder 1.3 (Carattoli, 2009)*.

The four bovine STEC O104:H7 strains carried 8 intact prophage sequences, whereas the bovine non-STEC strains carried only 1 to 5 intact prophage sequences. However, the number of intact prophage sequences in human STEC O104:H7 strains ranged from 2 to 10, with 2011C-3665 strain carrying 10 intact prophage sequences. The number of incomplete prophage sequences ranged from 9 to 12, 1 to 5, and 4 to 7 for bovine STEC, bovine non-STEC, and human STEC strains, respectively. Total number of questionable prophage sequences ranged from 1 to 4, 0 to 1, and 1 to 4 for bovine STEC, bovine non-STEC, and human STEC strains, respectively. One of the human non-STEC strains (07-3598) carried four intact, six incomplete and zero questionable prophage sequences (Table 4).

**Table 4:** Total number of prophage sequences in bovine and human *E. coli* O104 strains identified using PHASTER.

**Serotypes**	**Strains**	***stx***	**Completeness of prophage sequences^#^**		
			**Intact**	**Questionable**	**Incomplete**
**BOVINE**					
O104:H7	2013-6-685A	1c	8	1	9
O104:H7	2013-6-48C	1c	8	4	12
O104:H7	2013-6-122E	1c	8	3	10
O104:H7	2013-6-148B	1c	8	4	11
O104:H7	2013-6-193B	-	3	1	1
O104:H7	2013-6-289D	-	5	0	3
**HUMAN**					
O104:H7	06-3637	2d	3	4	4
O104:H7	08-4061	2a	2	3	7
O104:H7	2011C-3665	1c	10	1	7
O104:H7	2012C-3400	2a	5	1	5
O104:H7	07-3598	-	4	0	6
**BOVINE**					
O104:H8	2013-6-380B	-	5	0	5
O104:H12	2013-6-210A	-	1	0	3
O104:H23	2013-6-140D	-	3	0	3
O104:H23	2013-6-173D	-	4	0	1

# *Prophage sequences were classified as intact, questionable and incomplete based on the PHASTER scores >90, 70–90, and < 70, respectively (Zhou et al., 2011; Arndt et al., 2016)*.

### CRISPR-Cas system

The most common subtype of CRISPR-Cas system in most of the bovine and human *E. coli* O104 strains was I-A and I-E. Other subtypes identified were I-D (2011C-3665), I-F (2013-6-193B, 2013-6-289D), III-B (2013-6-148B), and VI-C (2013-6-148B). All the four bovine O104:H7 STEC strains and one the human O104:H7 STEC strain (2011C-3665) carried a single CRISPR locus, four human O104:H7 STEC strains and three *stx*-negative bovine strains carried two loci, and two *stx*-negative bovine strains carried four loci. One of the bovine O104:H12 strain (2013-6-210A) carried CRISPR region lacking *cas* genes. Total number, nucleotide sequence and average length of repeats and spacers were highly conserved across all *stx*-positive bovine O104:H7 STEC strains. Average length and nucleotide sequence of repeats and spacers of one of the human O104:H7 STEC strain (2011C-3665) was similar to bovine O104:H7 STEC strains, however, it carried fewer repeats and spacers compared to bovine O104:H7 STEC strains. Similarly, total number, nucleotide sequence and average length of repeats and spacers were highly conserved across all human O104:H7 STEC strains. Average length and nucleotide sequences of spacers and repeats were similar among bovine *stx*-negative O104:H7 strains, but they differed in number of repeats and spacer units. However, repeats and spacer units were not conserved among O104:H23 strains (Table 5).

**Table 5:** Characteristic features of CRISPR-Cas system in *E. coli* O104 strains isolated from cattle feces and human clinical cases^#^.

**Strains**	**Subtype**	**Cas Proteins**	**No. of loci**	**No. of repeats**	**Average length of repeats**	**No. of spacers**	**Average length of spacers**	Questionable CRISPR^*^
**CATTLE**								
2013-6-685A	I-E, I-A	Csa3, DEDDH, Cas3, Cas3 HD, Cas8e, Cse2gr11, Cas7, Cas6e, Cas1, Cas2, Cas5	1	11	29	10	32	+
2013-6-48C	I-E, I-A	Csa3, DEDDH, Cas3, Cas3 HD, Cas8e, Cse2gr11, Cas7, Cas6e, Cas1, Cas2, Cas5	1	11	29	10	32	+
2013-6-122E	I-E, I-A	Csa3, DEDDH, Cas3, Cas3 HD, Cas8e, Cse2gr11, Cas7, Cas6e, Cas1, Cas2, Cas5	1	11	29	10	32	+
2013-6-148B	I-E, I-A, III-B, VI-C	Csa3, DEDDH, Cas3, Cas3 HD, Cas8e, Cse2gr11, Cas7, Cas6e, Cas1, Cas2, Cas5, Cmr1gr7, C2c3	1	11	29	10	32	+
2013-6-193B	I-E, I-F	DEDDH, Cas8e, Cas6f, Cas7f, Cas5f, Cas8f, Cas3, Cas1. Cse2gr11, Cas7, Cas5, Cas6e, Cas1, Cas2, Csa3	4	19 (Locus 1) 15 (Locus 2) 10 (Locus 3) 16 (Locus 4)	28 (Locus 1) 28 (Locus 2) 29 (Locus 3) 29 (Locus 4)	18 (Locus 1) 14 (Locus 2) 9 (Locus 3) 15 (Locus 4)	32 (Locus 1) 32 (Locus 2) 32 (Locus 3) 31 (Locus 4)	-
2013-6-289D	I-A, I-E, I-F	Cas8e, DEDDH, Cas6f, Cas7f, Cas5f, Cas8f, Cas3, Cas1, Csa3, Cas3, Cas2, Cse2gr11, Cas7, Cas5, Cas6e	4	5 (Locus 1) 15 (Locus 2) 10 (Locus 3) 15 (Locus 4)	28 (Locus 1) 28 (Locus 2) 29 (Locus 3) 29 (Locus 4)	4 (Locus 1) 14 (Locus 2) 9 (Locus 3) 14 (Locus 4)	32 (Locus 1) 32 (Locus 2) 32 (Locus 3) 31 (Locus 4)	–
2013-6-380B	I-A, I-E	DEDDH, Cas8e, Cas3, Csa3, Cas1, Cas2, Cas6e, Cas5, Cas7, Cse2gr11	2	3 (Locus 1) 24 (Locus 2)	30 (Locus 1) 29 (Locus 2)	2 (Locus 1) 23 (Locus 2)	31 (Locus 1) 32 (Locus 2)	-
2013-6-210A	I-A, I-E	DEDDH, Csa3, Cas3, Cas8e	–	–	–	–	–	+
2013-6-140D	I-A, I-E	Csa3, DEDDH, Cas3, Cas2, Cas1, Cas6e, Cas5, Cas7, Cse2gr11, Cas8e	2	17 (Locus 1) 21 (Locus 2)	29 (Locus 1) 29 (Locus 2)	16 (Locus 1) 20 (Locus 2)	31 (Locus 1) 32 (Locus 2)	-
2013-6-173D	I-A, I-E	Cas3, Cas8e, Cse2gr11, Cas7, Cas5, Cas6e, Cas1, Cas2, Csa3, DEDDH	2	19 (Locus 1) 17 (Locus 2)	29 (Locus 1) 29 (Locus 2)	18 (Locus 1) 16 (Locus 2)	32 (Locus 1) 32 (Locus 2)	-
**HUMAN**								
06-3637	I-A, I-E	Csa3, DEDDH, Cas3, Cas8e, Cse2gr11, Cas7, Cas6e, Cas1, Cas2, Cas5	2	11 (Locus 1) 7 (Locus 2)	29 (Locus 1) 29 (Locus 2)	10 (Locus 1) 6 (Locus 2)	32 (Locus 1) 32 (Locus 2)	+
08-4061	I-E, I-A	Cas3, Cas8e, Cse2gr11, Cas7, Cas5, Cas6e, Cas1, Cas2, DEDDH, Csa3,	2	11 (Locus 1) 7 (Locus 2)	29 (Locus 1) 29 (Locus 2)	10 (Locus 1) 6 (Locus 2)	32 (Locus 1) 32 (Locus 2)	+
2011C-3665	I-A, I-D, I-E	Csa3, DEDDH, Cas3, Cas8e, Cse2gr11, Cas7, Cas5, Cas6e, Cas1, Cas2, Cas10d	1	9	29	8	32	+
2012C-3400	I-A, I-E	DEDDH, Cas8e, Cas3, Cse2gr11, Cas7, Cas5, Cas6e, Cas1, Cas2, Csa3	2	11 (Locus 1) 7 (Locus 2)	29 (Locus 1) 29 (Locus 2)	10 (Locus 1) 6 (Locus 2)	32 (Locus 1) 32 (Locus 2)	+
07-3598	I-E	Cas3. DEDDH, Csa3, Cas8e, Cse2gr11, Cas7, Cas5, Cas6e, Cas1, Cas2	2	11 (Locus 1) 7 (Locus 2)	29 (Locus 1) 29 (Locus 2)	10 (Locus 1) 6 (Locus 2)	32 (Locus 1) 32 (Locus 2)	+

# *Based on annotation by CRISPRone (Zhang and Ye, 2017)*.

* *Questionable CRISPR–Isolated CRISPR array (repeats and spacers) without cas genes*.

### Phylogenetic relationship and types (ST)

**Figure 1:**
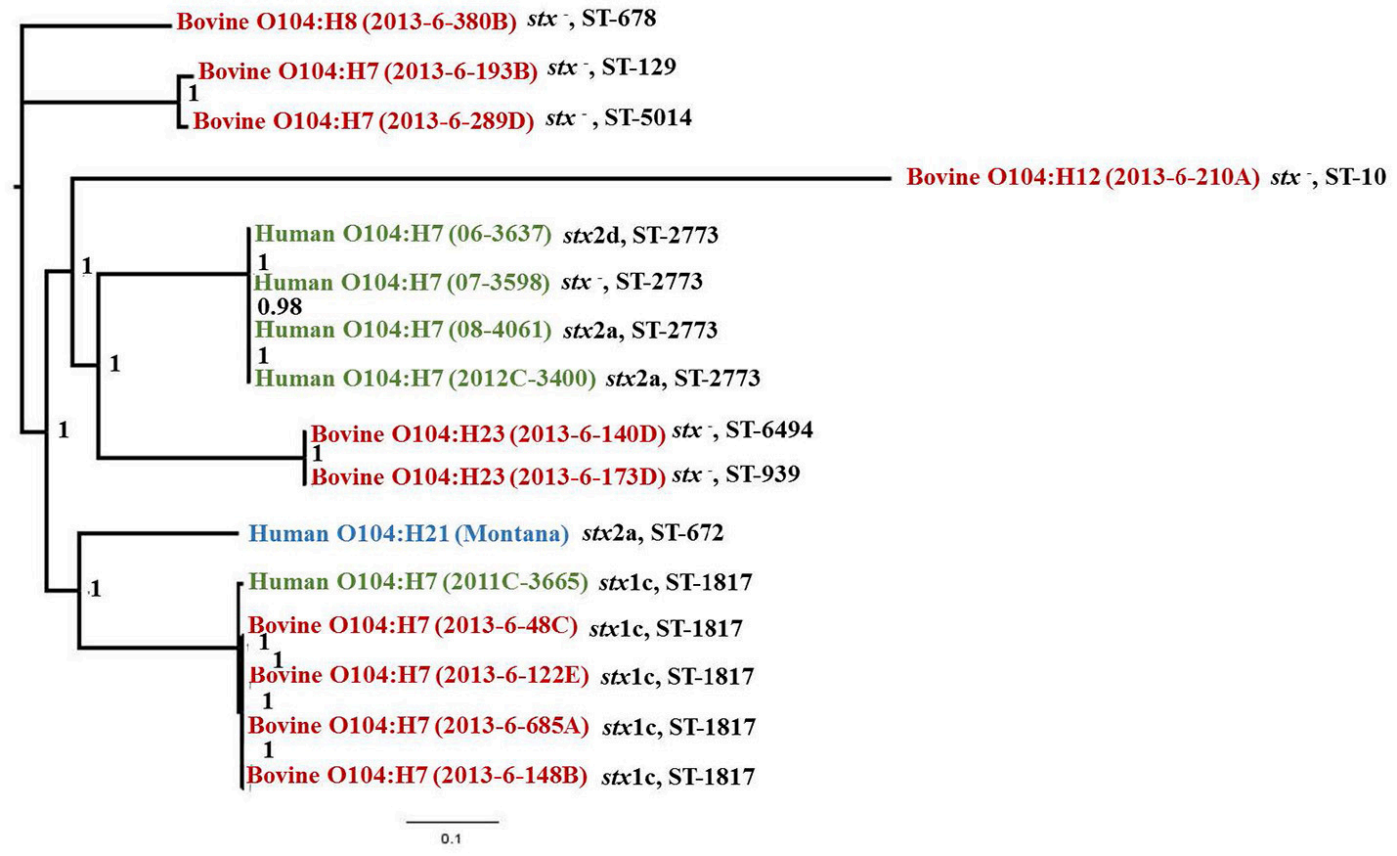
Phylogenetic tree and sequence types (ST) of *stx-*positive or –negative *E. coli* O104 strains isolated from cattle feces and human clinical strains constructed using Parsnp v1.2 and visualized using FigTree 1.4.3 (bootstrap values are provided for each branch).

Bovine *E. coli* O104 strains carrying the same flagellar type clustered together (Figure 1). Among bovine O104:H7 strains, STEC strains clustered separately from non-STEC strains. Additionally, the O104:H8 strain (2013-6-380B) was more closely related to non-STEC O104:H7 strains (Figure 1). *Escherichia coli* O104:H12 strain (2013-6-210A) was distantly related to other O104 serotypes. All human O104:H7 strains, except one, clustered together. The human O104:H7 strain, 2011C-3665, which carried *stx*1c gene similar to bovine STEC O104:H7 strains clustered with the bovine O104:H7 STEC strains. The human Montana outbreak strain of O104:H21 (Montana outbreak) clustered more closely with one of the human (2011C-3665) and bovine O104:H7 STEC strains (Figure 1).

The four bovine O104:H7 STEC strains and one of the four human O104:H7 strains (2011C-3665), which clustered with the bovine strains, belonged to ST-1817. The other 4 human O104:H7 strains that clustered separately belonged to ST-2773. One non-STEC bovine O104:H7 strain (2013-6-289D) belonged to ST-5014. The remaining bovine non-STEC O104 strains belonged to ST-678, ST-10, ST-6494, ST-129, and ST-939 (Figure 1).

## Discussion

Shiga toxin-producing *E. coli* O104 serotypes, other than O104:H4 and O104:H21, have been reported to be associated with sporadic cases of diarrhea in humans (Hussein, 2007; Miko et al., 2013). Only one small outbreak of hemorrhagic colitis in Montana, USA, has been reported with the O104:H21 serotype (CDC, 1995). Several studies have characterized the strains belonging to various O104 serotypes by PCR-based detection of specific virulence targets (Miko et al., 2013; Shridhar et al., 2016). However, there has been one study on the genome-scale analysis of *E. coli* O104 serotypes, other than the German outbreak serotype of O104:H4 (Lambert et al., 2015; Yan et al., 2015). Genomic characterization of *E. coli* O104:H4 German outbreak strain has revealed that the hybrid pathotype evolved by gain and loss of chromosomal and plasmid encoded virulence genes (Mellmann et al., 2011; Rasko et al., 2011). *Escherichia coli* O104:H4 has been reported to be evolved from EAEC by acquisition of *stx*2 prophage and plasmid encoding CTX-M-15 extended spectrum beta lactamase (Rohde et al., 2011). Yan et al. (2015) sequenced the genomes of two human strains of *E. coli* O104:H21 (Montana outbreak) and an O104:H7 STEC strain isolated from cattle and compared them to *E. coli* O104:H4 (German and Central Africa outbreak strains) strains. They reported that the O104:H7 STEC strain was more similar to the Montana strain (O104:H21) than to the German strain (O104:H4). To our knowledge, this is the first study to analyze the whole genome sequences of human O104:H7 strains and to compare the virulence gene profiles of bovine O104:H7 strains.

Genomes of *stx* carrying bovine O104:H7 strains were larger than the *stx*-negative strains of O104:H7 or other serotypes. The genome sizes were proportional to the number of genes carried on the mobile genetic elements (phages, prophages, transposable elements and plasmids). Yan et al. (2015) have attributed the variations in the genomes of the three O104 strains (two H21 and one H7) analyzed to the gain or loss of mobile genetic elements (Yan et al., 2015). Although results of our study suggested that the variation in the size of genomes is due to variation in the mobile genetic elements, variations in sequencing coverage cannot be ruled out.

Of all the bovine O104 serotypes (H7, H8, H12, and H23) that were included in the study, only *E. coli* O104:H7 serotype possessed Shiga toxins. Two human O104:H7 strains carried *stx*2 subtype a, and one of the strains carried *stx*2 subtype d. Shiga toxin 2a and 2d have been reported to be most commonly associated with severe human illness and complications, such as HUS (Bielaszewska et al., 2006; Persson et al., 2007; Iyoda et al., 2014). The German outbreak strains of O104:H4 possessed *stx*2a (Scheutz et al., 2011), whereas the Montana strain O104:21 carried *stx*2d (Yan et al., 2015). Four of the bovine and one of the human O104:H7 strains carried *stx*1 subtype c. Friedrich et al. (2003) reported that *stx*1c carrying STEC strains were isolated from asymptomatic patients and patients with uncomplicated diarrhea (Friedrich et al., 2003). However, a *stx*1c carrying *E. coli* O78:H- strain was isolated from a 2-week old boy suffering from bacteremia and HUS and from family members who were asymptomatic (Lienemann et al., 2012).

All the bovine and human strains included in our study were negative for intimin and other LEE-encoded virulence genes, and non-LEE encoded effectors. Absence of *eae* has been reported to be a feature of O104 human outbreak strains (O104:H4 German outbreak and O104:H21 Montana outbreak strains) (Feng et al., 2001; Scheutz et al., 2011). A significant association between strains that carry *stx*1c and LEE-negative strains has been reported (Haugum et al., 2014). Shiga toxin-producing *E. coli* strains negative for LEE-encoded virulence genes have been isolated from humans with HUS (Bonnet et al., 1998; Paton et al., 1999). Both human and cattle *E. coli* O104 strains carried other adhesins such as *iha* and *lpf* A. Long polar fimbriae (*lpf*) and *iha* that encode for *Irg*A homologous adhesins were the most prevalent adhesins identified in LEE-negative *E. coli* strains isolated from cattle and human sources (Galli et al., 2010). These adhesins (*iha* and *lpfA*) were also carried by *E. coli* O104:H4 German outbreak strains and *E. coli* O104:H21 Montana outbreak strain (Bielaszewska et al., 2011; Yan et al., 2015). Additionally, none of the bovine and human strains investigated in our study was positive for *efa*1 (enterohemorrhagic *E. coli* factor for adherence), which is not surprising because previous studies have suggested a strong association between *eae* and *efa*1 genes (Galli et al., 2010). Human O104:H7 strains, and not bovine O104:H7, were positive for subtilase cytotoxin, which has been reported to be involved in damaging human microvascular endothelial cells (Amaral et al., 2013). The *subA* gene has also been detected in *eae* negative *E. coli* O103:H21 strains associated with sporadic cases of HUS (Paton et al., 1999). One of the *stx* negative bovine O104 strains was positive for *ast*A gene, which encodes for EAEC heat-stable enterotoxin 1. Typical and atypical enteropathogenic *E. coli* strains carrying *astA* gene have been reported to be frequently associated with diarrhea (Silva et al., 2014).

Both bovine and human *E. coli* O104 strains included in the present study were positive for plasmid encoded virulence genes such as *ehx*A, *esp*P, *kat*P (encoded by pO157), and *epe*A (encoded by pO113). Enterohemolysin, encoded by *ehxA*, has been reported to be involved in increased production of IL-1β and cytotoxicity (Zhang et al., 2012). It has also been reported to be frequently associated with *E. coli* O111 STEC strains isolated from patients with HUS (Schmidt and Karch, 1996). Extracellular serine protease (*esp*P) is responsible for mucosal hemorrhage observed in patients suffering from hemorrhagic colitis, due to cleavage of pepsin A and human coagulation factor V (Brunder et al., 1997). An autotransporter protease, *epe*A has been reported to be frequently found in association with LEE-negative STEC strains. The protease is involved in adherence and colonization of small intestine due to its mucinolytic activity (Leyton et al., 2003). One of the bovine non-STEC O104:H7 strain (2013-6-193B) carried *pic* (protein involved in intestinal colonization), which encodes for serine protease autotransporter. The protease, also secreted by *Shigella flexneri* and enteroaggregative *E. coli*, is involved in intestinal colonization (Henderson et al., 1999). The *pic* gene was also carried by *E. coli* O104:H4 (German outbreak strain) (Bielaszewska et al., 2011). Other genes characteristic of EAEC such as *aggA* (pilin subunit of aggregative adherence fimbriae I), *agg3A* (pilin subunit of aggregative adherence fimbriae III), *aggR* (transcriptional regulator), and serine protease autotransporter toxins (*sepA; Shigella* extracellular protein A, *sigA; Shigella* IgA protease-like homolog, *aap;* dispersin, *aatPABCD;* dispersin transporter) were present in *E. coli* O104:H4 German outbreak strains, but absent in *E. coli* O104:H21 Montana outbreak strain (Bielaszewska et al., 2011; Frank et al., 2011; Yan et al., 2015) and *E. coli* O104 strains investigated in our study.

IncF plasmids (1B and II) were present in all bovine O104 STEC strains, except one (*stx* negative 2013-6-193B). Antimicrobial resistance genes, such as *bla*_TEM−1_, *bla*_OXA−1_, and *aac(6*′*)-Ib-cr*, are carried on IncF plasmids (Carattoli, 2009; Cergole-Novella et al., 2010). However, none of the bovine strains analyzed in the study carried antimicrobial resistance genes according to the ResFinder data base. The human O104:H7 strain (2011C-3665) that carried the three antimicrobial resistance genes (*tet, aadA1*, and *sul1*) was negative for IncF1, but positive for IncFII plasmids. Tetracycline resistance was the most common resistance in *E. coli* O157 strains isolated from bovine and human sources (Wilkerson et al., 2004). Non-O157 STEC serogroups isolated from human, bovine and ovine sources were found to be most commonly resistant to streptomycin, sulfisoxazole, and tetracycline (Wang et al., 2016). IncH12 plasmid, which was present in bovine strains, has been shown to be involved in the dissemination of antimicrobial resistance genes (Fernández et al., 2007).

Mobile genetic elements have been reported to influence pathogenicity, survival and spread of O157 and non-O157 EHEC (Ogura et al., 2009). CRISPR-Cas system provides acquired immunity against viruses and plasmids by targeting foreign nucleic acids (Horvath and Barrangou, 2010). Plasmid and phage environment have been reported to influence spacer composition (Delannoy et al., 2015a). Hence, we also analyzed the CRISPR-Cas systems in addition to phage and plasmid profiles of the *E. coli* O104 strains. Analysis of CRISPR-Cas system in bovine and human *E. coli* O104 strains revealed that it was highly conserved among O104:H7 bovine and human STEC strains. It was also conserved among *stx*-negative bovine O104:H7 strains. These findings show that the CRISPR systems are specific for serotype, regardless of the presence or absence of *stx*. Similar findings have also been reported in previous studies (Delannoy et al., 2012a,b; Toro et al., 2014). However, additional strains should be analyzed to show the serotype specificity of CRISPR loci in other O104 serotypes investigated in this study.

Analysis of phylogenetic relationship of O104 strains revealed that O104:H21 strain was more closely related to *stx*1c-carrying O104:H7 strains of bovine and human origin. This is in agreement with the previous study (Yan et al., 2015). In our study, O104 strains with the same H-type clustered together. *Escherichia coli* serogroups carrying the same flagellar antigen have been hypothesized to share common ancestors (Ju et al., 2012). Miko et al. (2013) reported that the O104 STEC strains carrying the same H-types had similar virulence gene profiles and clustered together by PFGE analysis (Miko et al., 2013). The genetic diversity of different O104 serotypes could be attributed to gain and loss of mobile genetic elements (Yan et al., 2015). Additionally, bovine O104 STEC strains and one of the human STEC strain (2011C-3665) which clustered together belonged to same sequence type (ST-1817). This suggests that the bovine feces could be a source of human illness caused by *E. coli* O104:H7 serotype. One of the non-STEC O104 strains belonged to ST-678, which includes *E. coli* O104:H4 German outbreak strain (Mora et al., 2011).

Although, this study provides sufficient evidence for the virulence potential of bovine *E. coli* O104 strains, the conclusions are based on a limited number of strains. Hence, further analysis of additional O104 strains belonging to various serotypes and sources might provide strong evidence for the virulence potential and evolution of *E. coli* O104 strains. Also, the use of long read sequencing technologies such as PacBio will enable in-depth analysis of the virulence potential of the strains compared to short read technologies such as Illumina. A combination of short read and long read technologies have also shown to generate high quality sequences which allow in-depth analysis of strains (Delannoy et al., 2015b, 2017).

## Conclusions

Whole genome analyses of *E. coli* O104 strains of different serotypes provide an insight into their genetic diversity and pathogenic potential. Strains of serotype O104:H7, could be of public health concern, because they carry Shiga toxin gene and several other genes encoding virulence factors responsible for human illness. Interestingly, the five O104:H7 strains of ST-1817 (1 human and 4 bovine) were all positive for the *stx*1c gene and clustered together phylogenetically. In contrast, four human O104:H7 strains of ST-2773, positive for either *stx*2a, *stx*2d or no Shiga toxin genes, clustered together, but were apart from the bovine ST-1817 strains. Similarly, CRISPR loci of four of the bovine O104:H7 STEC strains and one of the human O104:H7 STEC strain (2011C-3665) were conserved. These findings suggests that bovine feces could be a source of human O104:H7 STEC strains. The WGS-based analysis of virulence genes suggests that bovine O104:H7 STEC has the potential to be a diarrheagenic pathogen in humans.

## Nucleotide sequence accession numbers

The whole genome shotgun sequences have been deposited at DDBJ/ENA/GenBank under the accession numbers NEKE00000000 to NEKS00000000.

## Author contributions

TN, JB, CE, and NS conceived and designed the experiments. PS, IP, JG, LN, and XS performed the experiments. PS, IP, and JG analyzed the data. PS, TN, JB, and IP wrote the paper.

### Conflict of interest statement

The authors declare that the research was conducted in the absence of any commercial or financial relationships that could be construed as a potential conflict of interest.
